# Ephrin regulation of palate development

**DOI:** 10.3389/fphys.2012.00376

**Published:** 2012-09-26

**Authors:** M. Douglas Benson, Maria J. Serrano

**Affiliations:** Department of Biomedical Sciences, Texas A&M Health Science Center Baylor College of DentistryDallas, TX, USA

**Keywords:** palate, ephrin, EMT, fusion, epithelium

## Abstract

Studies of palate development are motivated by the all too common incidence of cleft palate, a birth defect that imposes a tremendous health burden and can leave lasting disfigurement. Although, mechanistic studies of palate growth and fusion have focused on growth factors such as Transforming Growth Factor ß-3 (Tgfß3), recent studies have revealed that the ephrin family of membrane bound ligands and their receptors, the Ephs, play central roles in palatal morphogenesis, growth, and fusion. In this mini-review, we will discuss the recent findings by our group and others on the functions of ephrins in palatal development.

## Overview of palate development

**Figure 1:**
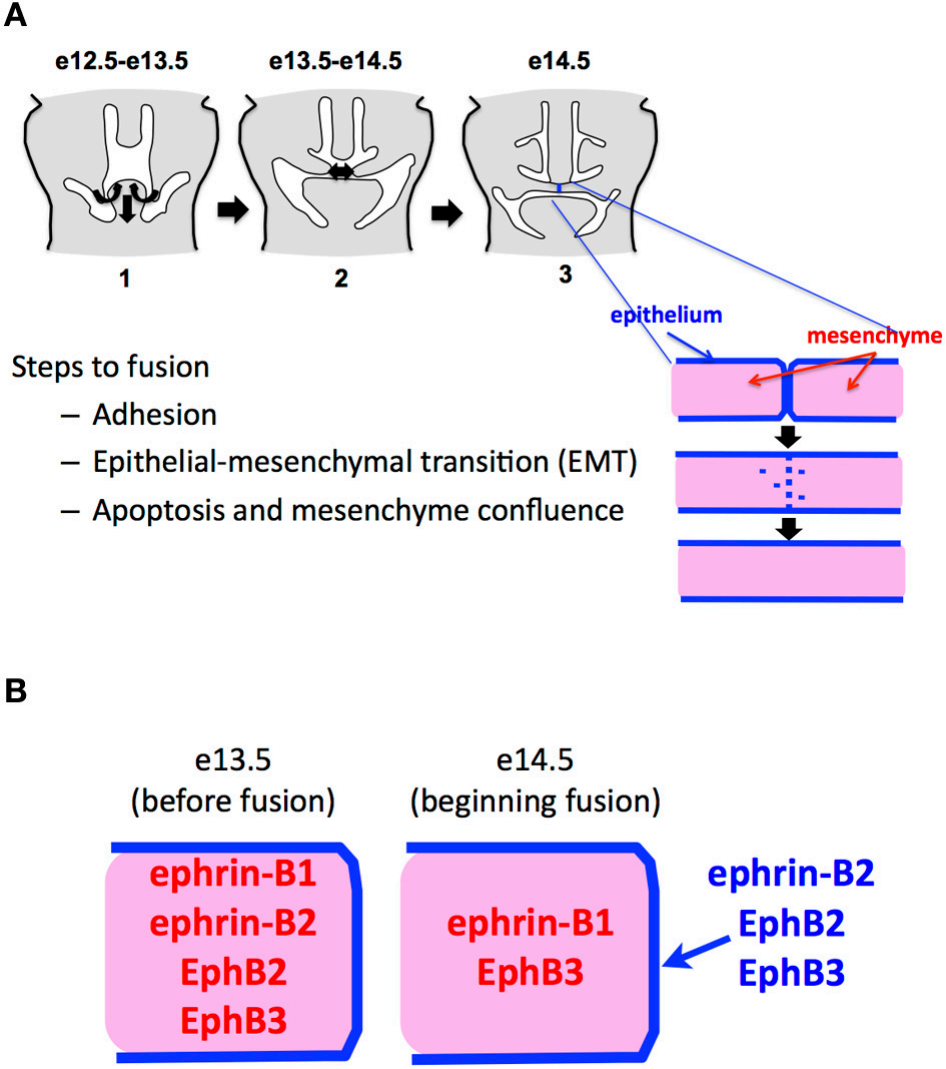
**Ephs and ephrins in fusing palate. (A)** Steps in mammalian palatal fusion. Palatal shelves of mesenchyme ensheathed in a two-cell thick epithelial layer elevate over the tongue and grow to midline. This happens beginning at about embryonic day 12.5 in the mouse. At e14.5, the epithelial cells adhere, migrate into the mesenchyme and/or die, leaving a confluent mesenchymal shelf. **(B)** Summary of published patterns of Eph and ephrin expression in the palate just before and during fusion.

The bony secondary palate forms the roof of the mouth and separates the oral and nasal cavities. In mammals, it originates as two shelves of cranial neural crest-derived mesenchyme that grow vertically on either side of the tongue and then elevate over the tongue to grow toward the midline. Fusion of the secondary palate requires the midline juxtaposition of the two-cell-thick epithelium that covers the palatal shelves. Upon contact, the outer periderm layer on each shelf sloughs off, and the basal epithelial layers from each shelf adhere to form the midline epithelial seam (MES), which becomes stabilized by desmosomal junctions between the adhering cells. The MES then degrades to leave a confluent mesenchymal shelf that ultimately ossifies (Nawshad, 2008) (Figure 1A). The fate of the MES cells during fusion has long been a source of controversy. One theory has the cells dying by apoptosis (Glücksmann, 1965; Martínez-Alvarez et al., 2000; Cuervo and Covarrubias, 2004), while another says they undergo epithelial-to-mesenchymal transition (EMT) and migrate into the surrounding mesenchyme (Fitchett and Hay, 1989; Shuler et al., 1991, 1992; Kang and Svoboda, 2002, 2005). Current evidence on the mechanism of MES degradation best fits a model in which the medial edge epithelial (MEE) cells undergo EMT, followed by apoptosis (Ahmed et al., 2007).

For most of two decades, it has been understood that MEE degradation and palatal fusion requires Tgfß3. In mammalian palates, which normally fuse on their own, this factor is produced by the palatal tissue itself (Proetzel et al., 1995; Taya et al., 1999), and genetic ablation of Tgfß3 results in cleft palate. Chicken palates, which do not normally fuse, can be induced to fuse by adding exogenous Tgfß3, thus showing that they retain the signaling machinery to respond to this factor and validating the chicken as a useful model system to study palate development (Sun et al., 1998). In addition to the apparent requirement for diffusible Tgfß3, there is a contact-mediated signal that is also needed to initiate fusion. Palatal shelves (chicken or mouse) will not degrade their epithelial layers unless placed in direct contact, even when supplied with exogenous Tgfß3 (Sun et al., 1998). Recent studies suggest that this contact-mediated signal may be supplied by members of the ephrin family of membrane-bound ligands.

## Ephrin involvement in palatal growth and fusion

The Eph family is the largest family of mammalian receptor tyrosine kinases. Ephs and their membrane-bound ephrin ligands are responsible for multiple adhesion, migration, and boundary-forming events throughout development, particularly midline fusion events such as urorectal closure (Kullander and Klein, 2002; Murai and Pasquale, 2003; Dravis et al., 2004). Binding of ephrins to Ephs on opposing cells causes kinase activation in the Eph-bearing cells (forward signaling), while binding of Ephs can activate intracellular signaling inside ephrin-bearing cells (reverse signaling). Ephrin-Bs are transmembrane proteins that have conserved intracellular signaling domains while ephrin-As are glycosylphosphatidyl-inositol (GPI)-linked and use co-receptors to signal. Ephrin-As preferentially bind to the EphA subclass of receptors, while ephrin-Bs bind to EphBs, although there is physiologically relevant binding across classes, most notably between EphA4 and all three B ephrins.

Over the years, a number of genetically modified Eph and ephrin alleles have been created in mice to both track expression of these molecules and to examine the roles of forward and reverse signaling in developmental processes. In addition to traditional gene knockouts, several LacZ knock-in alleles have been generated. In these, either the entire protein or just the cytoplasmic domain of the Eph or ephrin-B in question is replaced with a bacterial beta-galactosidase moiety that can be visualized in tissue by incubation with X-gal to produce a blue precipitate. The chimeric alleles are especially useful because they lack intracellular signaling ability while retaining activity as ligands from their extracellular domains. Thus, they can be used to dissect forward and reverse ephrin signaling pathways.

The first evidence that ephrins play a role in palate development came with the linkage of 26 separate ephrin-B1 mutations to craniofrontonasal syndrome in humans, of which cleft palate is a prominent feature (Twigg et al., 2004; Wieland et al., 2005). At the same time, Davy, et al. reported that deletion of ephrin-B1 in cranial neural crest cells in mice caused craniofacial deformities, including cleft palate (Davy et al., 2004). The fact that these defects resulted from cell-autonomous ephrin-B1 deletion suggested that ephrin-B1 reverse signaling is important for palate formation. Five years later, Risley et al. reported that forward signaling through the combination of EphB2 and EphB3 is necessary for growth of palatal mesenchyme (Risley et al., 2009). These authors used EphB2 LacZ/LacZ; EphB3 -/- compound mutant mice to create forward signaling double knockout mice (EphB3 signaling is removed while EphB2 forward signaling is removed and reverse signaling is still intact). These mice had cleft palate from stunted palatal shelf growth, while EphB2 and EphB3 single mutants alone did not. Shortly after the Risley study, Bush et al. found that forward signaling from ephrin-B1 in palate mesenchyme was required for mesenchymal proliferation through a mechanism requiring MAPK/ERK activation. Without ephrin-B1, mice displayed cleft palate because the shelves failed to grow to midline (Bush and Soriano, 2010). These data together suggest that Ephs B2 and B3 function as the receptors for ephrin-B1 in palate mesenchyme. The EphB2 kinase was recently shown to increase proliferation in intestinal cypts through stimulation of Cyclin-D1 levels downstream of Abl activation (Genander et al., 2009). It will be interesting to see if this mechanism is also at play involved in palatal mesenchyme and is thus part of a more generalized program of EphB regulated proliferation in development.

When palatal shelves from EphB2 LacZ/LacZ; EphB3 -/- compound mutants were placed in contact with each other in culture, they adhered to form an MES and fused normally (Risley et al., 2009). This demonstrated that EphB2 and EphB3 forward signaling are not required for fusion, and that reverse signaling from EphB3 alone is not critical for fusion, although the extracellular domain of EphB2 was still able to act as a ligand for reverse signaling in these mice.

We examined in embryonic palate the expression of Ephs and ephrins for which we had LacZ indicator mouse lines. A summary of these expression patterns in fusing palate combined with those for Ephs and ephrins in the published literature is presented in Figure 1B. We found that ephrin-B2 and EphB2 were expressed specifically in the MES immediately prior to and during its degradation. This suggested to us that ephrin signaling contributes to palatal EMT and fusion. We found that by adding EphB2/Fc chimeric protein clustered with anti-Fc (Ephs and ephrins must be aggregated into clusters of at least four to have biological activity as ligands), we could cause fusion in chicken palates, even without adding the Tgfß3 that is normally required in chicken for fusion. This confirmed that EphB2 can indeed act as a ligand to induce fusion. Further, we observed that addition of unclustered EphA4/Fc protein, which promiscuously binds all B-ephrins without activating signaling and thus acts as a competitive inhibitor, blocked fusion even in the presence of Tgfß3. We also applied unclustered EphA4/Fc to mouse palates in culture and effectively blocked their fusion. Together, these experiments demonstrated that ephrin-B reverse signaling is necessary and sufficient for palate fusion (San Miguel et al., 2011).

Shortly following publication of our findings, Dravis et al. reported a study in which 26% of mice homozygous for the ephrin-B2/LacZ allele had cleft palate (Dravis and Henkemeyer, 2011). While this supports our findings, the fact that only a minority of these embryos had cleft palate, suggests that other Eph and ephrin family members contribute to reverse signaling at the midline and remain unaccounted for. This is not surprising, as Eph/ephrin mediated developmental processes are frequently under redundant control by multiple family members, including in palate, as noted above. We used our *ex vivo* palate culture system to examine fusion ability of palatal shelves from ephrin-B1 and ephrin-B2 classical knockout mice and found that both single knockouts fused. However, both showed deficiencies in medial and posterior fusion compared to wild type littermate controls. This suggests a delay in fusion caused by these knockouts (Maria J. Serrano and M. Douglas Benson unpublished observations). Unfortunately, we were unable to generate double knockout embryos to examine the effect of combined ephrin-B1 and -B2 abrogation. Interestingly, the study by Dravis et al. also showed ephrin-B2 expression in the mesenchyme before its re-localization to the MEE at the time of fusion, suggesting that ephrin-B2 plays a role in palatal shelf growth alongside ephrin-B1. They also showed EphB3 expression in the MEE at fusion, implicating it in the fusion process. These data emphasize the likely involvement of multiple Eph and ephrin family members in both phases of palatal development.

If ephrin-B1 is expressed in the palatal mesenchyme, and ephrin-B2 in the epithelium, how might these two molecules combine to mediate MES degradation and fusion? One possible answer may be found in recent studies on the role of ephrin signaling in cancer cell migration. Astin et al. demonstrated that prostate cancer cells are prodded along in their migration through fibroblasts by the activation of EphB3/EphB4 forward signaling in response to ephrin-B2 ligand from the surrounding fibroblasts (Astin et al., 2010). This forward signal activates Cdc42 within the cancer cells to eliminate contact inhibition and increase their invasiveness. It may be that a similar mechanism is at work in palatal MEE cell migration through the ephrin-B1-expressing mesenchyme. Whereas reverse signaling in MEE cells initiated by contact with Ephs (acting as ligands in reverse signaling) on the opposing shelf MEE begins the process of EMT, mesenchymal ephrin-B1 (acting as ligand) activates forward signaling in the former epithelial cells to continue their migration and complete MES degradation. Ephrin-B1 may also provide a signal to the migrating former MEE cells that causes their eventual apoptosis, as B ephrin forward signaling is known to cause apoptosis in other systems (Davies et al., 2009).

## Ephrin signaling in palatal EMT and fusion

As described above, we found that activation of ephrin reverse signaling in the chicken palate is sufficient to cause palatal fusion without the presence of Tgfß3, and that Tgfß3 cannot cause fusion without the ephrin signal. Yet there is clearly a question of signaling level. The fact that chicken palates cultured without Tgfß3 will not fuse unless exogenous EphB2/Fc is added, and that Tgfß3 knockout mouse palates do not normally fuse, indicates that the level of ephrin reverse signal naturally present in palatal tissue is not enough to overcome a lack of Tgfß signaling. The Tgfß3 and ephrin pathways must interact in one of two ways. The first possibility is that Tgfß3 activates expression of ephrins and/or Ephs in palate tissue to reach a threshold level required to activate fusion. In this model, ephrins are genetically and mechanistically downstream of Tgfß3. The second is that the two act in parallel, but intersect such that the Tgfß3-activated signals add to those elicited by ephrin activation to reach the level necessary to cause MES degradation. The ephrin signal must still be preeminent; however, as elevated ephrin stimulation obviated the need for Tgfß3 in our palate fusion assay, while addition of exogenous Tgfß3 did not compensate for a lack of ephrin signal. The activity of phosphatidylinositol 3-kinase (PI3K) is required for Tgfß3 stimulation of fusion (Kang and Svoboda, 2002), and our recent study discovered that the same is true for ephrin reverse signaling (San Miguel et al., 2011), as palates stimulated in culture with EphB2/Fc did not fuse in the presence of the PI3K inhibitor LY294002.

Our data on ephrin-B2 expression supports the EMT model of palatal fusion in that we observed the cells of the ephrin-B2-positive MES in the act of dispersing into palatal mesenchyme during fusion. Epithelial cells have a polarized, inflexible morphology maintained by specific networks of intermediate filaments, cell-cell junctions, and adhesions to the extracellular matrix. The transition to a more fibroblastic, motile phenotype such as is observed in the palatal MES, requires the dismantling of these networks in favor of a more fluid cytoskeletal arrangement and more plastic cell-cell contacts. Cytokeratin intermediate filaments disappear in favor of vimentin, laminin-1 content in the extracellular matrix decreases as fibronectin increases, and E-cadherin based adherins junctions are replaced by N-cadherin based cell-cell contacts (Yu et al., 2009; Jing et al., 2011). These changes in expression are governed by a set of transcription factors such as Twist1 and Snail, both of which are regulators of EMT during gastrulation and palate development (Yu et al., 2008, 2009; Qin et al., 2012). Thus, EMT involves both a reorganization of the cytoskeleton and a major shift in gene expression. So, how do ephrins contribute to these events?

**Figure 2:**
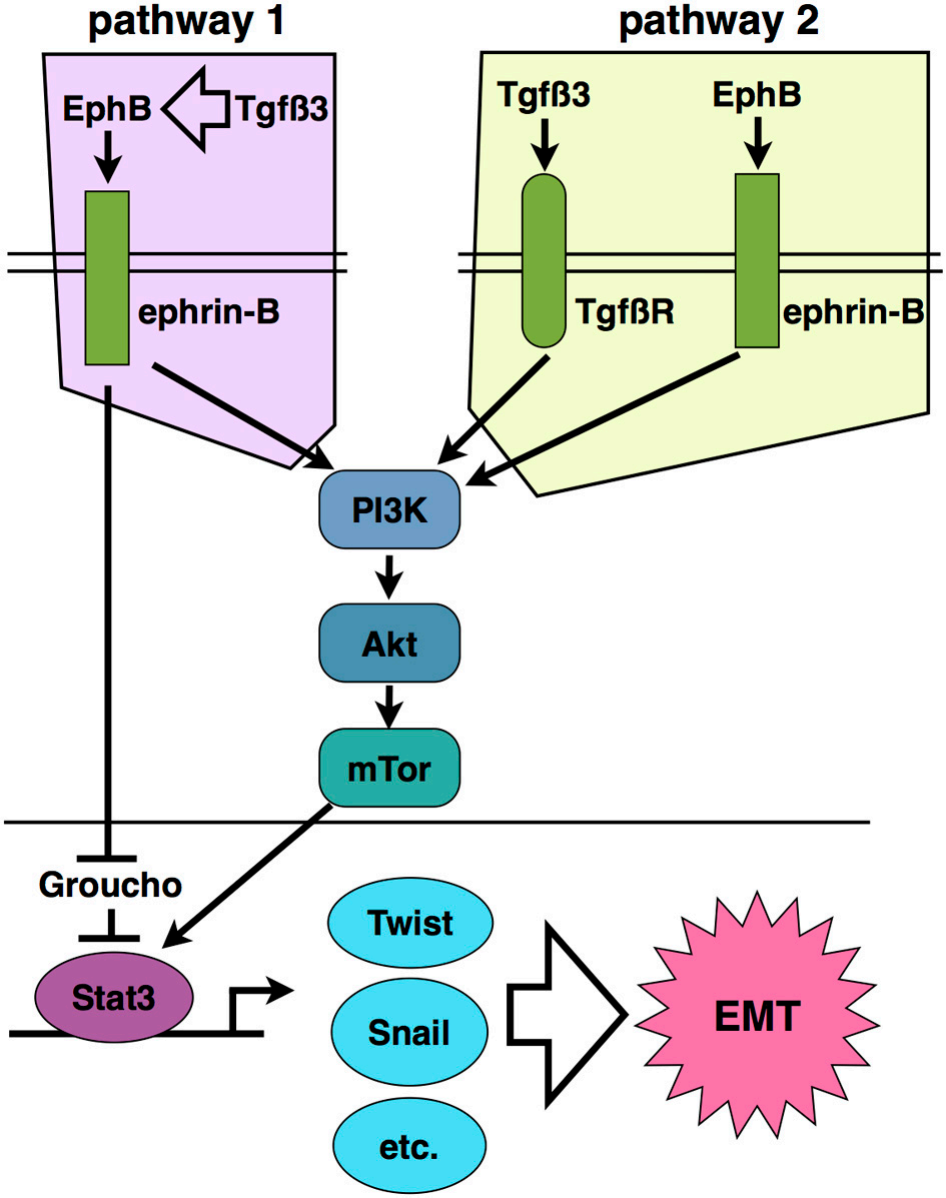
**Two proposed models of ephrin-B reverse signaling in palatal EMT based on current evidence.** Pathway 1 places ephrin signaling downstream of Tgfß3 signaling such that Tgfß3 stimulates expression of EphBs and/or ephrin-Bs, leading to activation of PI3K signaling. In pathway 2, Tgfß3 and ephrin-B signaling act in parallel to stimulate PI3K together. Pathways 1 and 2 are not mutually exclusive.

Part of the answer is found in the EMT that is required for metastasis of epithelia-derived tumors (Thiery, 2002). In certain settings, repulsion between Ephs and ephrins serves to keep potentially cancerous cells within their niche, such as in the colon, where ephrins keep intestinal crypt stem cells from migrating to the luminal ends of villi to form tumors (Holmberg et al., 2006; Genander et al., 2009). In instances such as these, Ephs appear to function as tumor suppressors. In many other cases; however, ephrins are upregulated in cancers, and their expression is associated with increased EMT and metastasis of malignancies. As mentioned above, the study by Astin et al. demonstrated that ephrin reverse signaling enables the loss of contact inhibition seen in prostate cancer cells and promotes their migration past normal fibroblasts (Astin et al., 2010). Our novel finding of PI3K involvement in ephrin reverse signaling provides a connection to this migration mechanism. PI3K signals to Akt, which activates the mTor complex, leading to migration of cancer cells. This pathway is frequently activated in malignancies, and inhibition of the mTor complex proteins Raptor and Rictor retards cancer cell invasiveness and suppresses the EMT required for metastasis (Gulhati et al., 2011). This mechanism may control the EMT and migration of epithelial cells during palatal fusion (Figure 2).

The PI3K/Akt/mTor system also connects to transcriptional activation associated with cancer EMT. The mTor kinase phosphorylates the signal transducer and activator of transcription 3 (Stat3) on Ser727, and thereby activates a transcriptional program of growth and invasiveness (Yokogami et al., 2000; Zhou et al., 2007). Stat3 activation is frequently associated with carcinoma invasiveness and poor prognosis (Yue et al., 2012). Active Stat3 upregulates Twist1 and Snail, which in turn suppress E-cadherin expression (Yamashita et al., 2004; Qin et al., 2012). Svoboda et al. demonstrated that Twist1 regulates palatal fusion (Yu et al., 2008). Thus, PI3K potentially connects ephrin-B reverse signaling to an EMT-associated gene expression program in palate. Phosporylated ephrin-B1 was also reported to bind directly to Stat3 in embryos and tumor cells, suggesting that direct recruitment of this transcription factor to the cytoplasmic domain of ephrin-Bs contributes to its activation (Bong et al., 2007). In addition to being a transcriptional activator, the ephrin-B1 cytodomain has been shown to bind the transcriptional repressor Groucho/TLE (Kamata et al., 2011). Though the significance of this binding to EMT is unknown, Groucho has been reported to repress transcription downstream of Tgfß signaling, thus providing another potential cross-interaction with the Tgfß3 system in palate (Hasson and Paroush, 2006).

## Conclusion

The study of ephrins in palate development is still in its infancy. We now know that ephrin forward signaling is necessary for early palatal shelf growth, and that ephrin reverse signaling is required for fusion of those shelves. Important questions remain, such as: (1) which Ephs and ephrins control fusion, (2) what are the specific downstream effectors of Ephs and ephrins in palatal mesenchyme and epithelium, (3) how do Tgfß3 and ephrin signaling pathways intersect, and (4) what elements of the transcriptional program in palatal EMT are controlled by ephrin signaling? The large collection of molecular and genetic tools available for studying ephrins in development makes it certain that efforts to answer these questions will accelerate in the coming years, and this will benefit both the fields of craniofacial biology and cancer.

### Conflict of interest statement

The authors declare that the research was conducted in the absence of any commercial or financial relationships that could be construed as a potential conflict of interest.
